# Perspectives of Physical Therapists in Saudi Arabia on radiological interpretation: attitudes, engagement, and educational needs

**DOI:** 10.1186/s12909-025-08367-1

**Published:** 2025-12-02

**Authors:** Samia A. Alamrani, Ali H. Alghamdi, Ahmad A. Alharbi, Hamad S. Al Amer, Abdulaziz A. Albalwi, Aisha I. Alasiri, Ibrahim E. Alfaifi

**Affiliations:** 1 Department of Health Rehabilitation Sciences, Faculty of Applied Medical Sciences, University of Tabuk, Tabuk, 71491 Saudi Arabia; 2 Department of Radiological Sciences, Faculty of Applied Medical Sciences, University of Tabuk, Tabuk, 71491 Saudi Arabia; 3 General Administration of Medical Services, University of Tabuk, Tabuk, 71491 Saudi Arabia

**Keywords:** Physical therapists, Radiology, Diagnostic imaging, Education, Professional

## Abstract

**Background:**

Radiological imaging is essential in clinical practice to support diagnosis and treatment planning. As Physical Therapists (PTs) increasingly collaborate within multidisciplinary teams, their ability to interpret radiographs has become more relevant. In Saudi Arabia, limited data exist concerning the involvement of PTs in radiological interpretation. Therefore, this study aimed to explore PTs’ engagement with radiological information, assess their attitudes, and examine the factors influencing their involvement in and interest in imaging education.

**Methods:**

This cross-sectional descriptive study employed a self-structured questionnaire to gather data on demographics, professional characteristics, practice patterns, learning sources, perceived barriers, and attitudes. Chi-square tests were used to assess associations, and binary logistic regression was used to identify predictors of interest in further education.

**Results:**

Among the 241 PTs surveyed, 46.1% reported frequent involvement in radiological interpretation, and 83.0% believed it should be part of their professional role. Academic education was the main learning source, while 40.0% identified insufficient training as a key barrier. Engagement levels and attitudes were significantly associated with qualification, experience, workplace setting, and specialization. Notably, PTs who rarely contributed were four times more likely to express interest in further education (OR = 4.0, 95% CI: 1.5–10.4, *p* = 0.007).

**Conclusion:**

Many PTs in Saudi Arabia reported engaging in radiological interpretation, though the extent and accuracy of these contributions remain self-reported rather than objectively confirmed. Their involvement was influenced by education, clinical experience, and workplace setting. The findings highlight the need to integrate imaging content into national curricula and continuing professional development programs. Enhancing these competencies has the potential to strengthen collaborative care and may contribute to improved clinical decision-making and healthcare outcomes.

**Clinical trial number:**

Not applicable.

**Supplementary Information:**

The online version contains supplementary material available at 10.1186/s12909-025-08367-1.

## Background

Radiological interpretation is an essential skill in modern clinical practice, supporting accurate diagnosis, guiding therapeutic decision-making, and promoting effective interdisciplinary collaboration [1, 2]. Interpretation of radiological images is usually the exclusive domain of physicians, radiologists, and diagnostic radiographers. However, the evolving scope of practice for Physical Therapists (PTs) in regions such as Saudi Arabia is reshaping standard boundaries in healthcare [3–5, 5]. This emerging clinical responsibility has stimulated growing interest in the competencies, accountability, and educational readiness of PTs regarding radiological interpretation. Previous studies have explored the integration of imaging into physical therapy education and practice, highlighting its potential to enhance clinical decision-making and improve patient outcomes. In some settings, this integration has helped PTs make more appropriate referrals by enabling them to better recognize when further imaging or medical consultation is needed [6–9]. The focus has largely been on referral patterns and regulatory frameworks, with the identified barriers including limited formal training in diagnostic imaging, inconsistent access to continuing education, and legal or institutional constraints [8]. Nonetheless, there remains a notable gap in the literature concerning the extent to which PTs actively engage in the interpretation of radiological images and how they perceive their role in this domain.

The importance of radiological interpretation in physical therapy practice is multifold. First, accurate interpretation of diagnostic images can lead to more precise treatment planning, especially for musculoskeletal conditions where incidental variations in imaging findings may influence therapeutic approaches [1, 10]. Second, as physical therapy practice in Saudi Arabia evolve toward greater autonomy, there is a growing need for professionals to develop competencies to determine the need for imaging and to interpret findings that inform clinical reasoning and patient management [5, 8]. Third, the integration of radiological interpretation into physical therapy practices may help reduce delays in patient management, thereby improving patient outcomes and reducing overall healthcare costs [6, 11].

For PTs managing orthopedic or movement-related conditions, timely radiological interpretation can increase care efficiency and reduce diagnostic delays, particularly in direct access or primary care models [4, 9, 12]. Globally, there is increasing momentum toward incorporating imaging competencies into physical therapy education and professional standards [13, 14]. Research from different countries has demonstrated that PTs can competently interpret certain types of radiological images when provided with appropriate training and guidelines [15, 16]. These developments have been supported by professional bodies such as the American Physical Therapy Association (APTA) and the Australian Physiotherapy Association (APA), both of which have recognized advanced imaging competencies and established pathways for advanced practice roles e.g. APTA Imaging Special Interest Group (I-SIG) [17]. However, there are substantial variations in the extent of the integration and acceptance of such practices between nations, institutional policy, and local scope-of-practice regulations [17, 18].

As healthcare systems in the region move toward greater efficiency and interdisciplinary care [19–21], understanding the current status of radiological competencies among PTs is vital for informing policy, curriculum development, and continuing education. In Saudi Arabia, physical therapy is a rapidly expanding profession, yet little is known about PTs’ engagement with radiological interpretation, their perceptions of its relevance, or the barriers that may limit their involvement. Moreover, no national-level data have been published to explore the frequency with which Saudi PTs contribute to radiograph interpretation or the factors that may influence these practices. A deeper understanding of these dimensions is essential for aligning professional competencies with modern clinical demands and advancing the quality of physical therapy care.

### Study aims

This study was designed to explore PTs’ engagement with radiological interpretation and assess their attitudes toward interpreting radiographs. Additionally, it aimed to examine the demographic and professional factors associated with their contribution to and interest in radiological interpretation and related educational activities.

## Methods

### Study design and ethical considerations

A cross-sectional descriptive design was chosen because it allows for the collection of data at a single point in time to explore existing attitudes, engagement levels, and associated factors among PTs. This approach is ideal for identifying prevalent characteristics and associations within the targeted population without manipulating variables or observing long-term trends. Ethical approval to conduct the study was obtained from the Local Research Ethics Committee at the University of Tabuk (UT-505-313-2025). Participants were informed that participation was voluntary, and they could withdraw at any time without consequences. All procedures were conducted in accordance with the ethical standards of the Declaration of Helsinki.

### Participants

Participants were recruited via a convenience sampling approach from different regions and professional sectors (public and private) in Saudi Arabia. On the basis of an estimated population of approximately 7,000 licensed PTs in the country [22], a sample size of 365 was calculated via Raosoft^®^ software , with a 95% confidence level and a 5% margin of error. Eligible participants included Saudi licensed PTs holding a recognized certificate in physical therapy, while physical therapy students, interns, and retired professionals were excluded.

### Informed consent

Electronic informed consent was obtained from all participants prior to completing the survey. An online information sheet was provided, detailing the study’s purpose, procedures, potential benefits and risks, and measures taken to ensure confidentiality. Consent was indicated by selecting the “agree” option on the electronic form. Participants were also given the opportunity to contact the research team via email to ask questions or seek clarification before providing their consent.

### Data collection procedure

Data collection was carried out between January 5, 2025, and May 2025, over a period of approximately 4 months. Participant invitations were distributed via institutional emails, professional networks, and social media platforms commonly used by PTs in Saudi Arabia, including X, *LinkedIn*, and *WhatsApp professional groups*. No incentives were offered for participation. The data were collected using a self-developed questionnaire specifically designed for this study, which was administered through the Google Forms platform. The full questionnaire is available in the Supplementary material (Appendix A).

The questionnaire consisted of three parts. The first part collected demographic information including sex, age, and region of practice. The second part focused on work-related characteristics such as qualifications, years of experience, workplace setting, and areas of specialization. The third part included items assessing PTs’ engagement with radiological interpretation, sources of learning, perceived barriers, attitudes toward the role of PTs in radiograph interpretation, and interest in related educational activities. The clarity and relevance of the questionnaire were assessed by a panel of 14 healthcare professionals including PTs and radiologists, via the item-level content validity index (I-CVI), the average scale-level content validity index (S-CVI/Ave), and the universal agreement index (S-CVI/UA) . The questionnaire was rated as clear and relevant, as illustrated in Table 1.

**Table 1:** Results of the content validity evaluation of the questionnaire used in the study (*n* = 14)

Variable	Clarity	Relevance
Number of Items with I-CVI ≥ 0.70	6	6
Number of Items with I-CVI < 0.70	0	0
Minimum–Maximum I-CVI	0.93-1.00	0.93-1.00
S-CVI/Ave	0.98	0.99
S-CVI/UA	0.67	0.83

*I-CVI* item-level content validity index, *S-CVI/Ave* average scale-level content validity index, *S-CVI/UA* universal agreement

As shown in Table 1, all the items scored above the minimum I-CVI threshold of 0.70, with results ranging from 0.93 to 1. This suggests that the items are both clear and relevant according to expert panel. The S-CVI/Ave scores also support this, showing strong agreement overall—0.98 for clarity and 0.99 for relevance. Additionally, the S-CVI/UA values indicate a high degree of consensus among the reviewers, further reinforcing the tool’s overall content validity.

### Data analysis

Statistical analyses were performed to assess participants’ frequency of contribution and attitudes toward radiograph interpretation. For the purpose of this study, the term *“contribution and attitudes toward radiograph interpretation”* was defined as any form of PTs' involvement in utilizing or applying radiological findings within clinical practice. This included: (i) discussing imaging findings with radiologists or referring physicians; (ii) reviewing and integrating information from radiology reports to guide assessment, clinical reasoning, and treatment planning; and (iii) using imaging findings to adjust management strategies or to support patient education and shared decision-making. Due to the structure of the dataset, it was not possible to distinguish precisely between these different forms of engagement; however, all were captured under the general category of *“contribution and attitudes toward radiograph interpretation.”*

The analyses also examined how demographic and professional characteristics were associated with frequent contributions and agreement with PTs interpreting radiographs. Additionally, the relationships between contribution frequency, beliefs, and interest in radiological interpretation and education were explored. For analysis purposes, “Never”, “Rarely”, “Often”, and “Always” responses on the frequency of radiological interpretation were recoded into “Rarely” and “Frequently”, respectively, whereas “Strongly disagree”, “Disagree”, “Agree”, and “Strongly agree” responses on attitudes toward radiograph interpretation were grouped into “Disagree” and “Agree”, respectively.

Chi-square tests were used to assess differences in contribution frequency and attitudes toward radiograph interpretation. When significant, pairwise comparisons were performed to identify specific group differences. Chi-square tests were also applied to evaluate the associations between demographic and professional characteristics and (1) frequent contributions to radiological interpretation and (2) agreement that PTs should interpret radiographs. Finally, binary logistic regression analyses were conducted to assess the relationships between contribution frequency, beliefs, and interest in radiological interpretation and education, with the results reported as odds ratios (ORs) and 95% confidence intervals (CIs). A sensitivity analysis was also conducted to examine the robustness of the logistic regression findings using alternative categorizations of key variables. For this purpose, “Rarely” and “Sometimes” responses for contribution frequency were combined and compared with “Frequently,” and “Disagree” and “Neutral” responses for attitudes toward radiograph interpretation were combined and compared with “Agree.” Logistic regression models were re-run using these alternative groupings to evaluate the direction or significance of the associations changed. All analyses were conducted using IBM SPSS Statistics for Windows, version 25.0 (Armonk, NY). Statistical significance was determined at an alpha level of 0.05.

## Results

### Participant characteristics

Table 2 summarizes the participants’ characteristics. A total of 241 PTs participated in the study. Of these, 54.8% were female, and 45.2% were male. Nearly half of the participants (49.8%) were aged 22–29 years, followed by 36.1% aged 30–39 years, 12.4% aged 40–49 years, and 1.7% aged 50–59 years. The participants were distributed across five regions in Saudi Arabia, with the highest representation from the central region (29.5%), followed by the northern (27.0%), southern (19.5%), western (16.2%), and eastern regions (7.9%). With respect to educational qualifications for physical therapy, the majority held a bachelor’s degree (68.0%), whereas others reported holding a master’s degree (12.0%), a doctoral degree (12.0%), a doctor of physical therapy degree (5.0%), or a diploma certificate (2.9%).

In terms of practice experience, approximately one-third (34.9%) had 1–5 years of experience, 20.3% had 6–10 years, 19.5% had 11–20 years, and 6.2% had over 20 years of experience. Approximately 19.1% had less than one year of experience. With respect to workplaces, 46.1% were employed in public hospitals or clinics, 35.7% were employed in private settings, 16.2% were employed in university-affiliated facilities, and 2.1% were unemployed. The most common area of specialization was general physical therapy (55.2%), followed by orthopedic (21.6%), pediatric (7.5%), neurological (5.0%), sports (4.1%), chest (2.1%), and geriatric (2.1%). Approximately 2.5% reported other specialties including women’s health, oncology, lymphoedema, and biomechanics or movement sciences.

**Table 2:** Characteristics of the participating PTs (*n* = 241)

Characteristic	*n*	%
Sex		
Male	109	45.2
Female	132	54.8
Age		
22–29 years	120	49.8
30–39 years	87	36.1
40–49 years	30	12.4
50–59 years	4	1.7
Practicing region in Saudi Arabia		
Central region	71	29.5
Western region	39	16.2
Eastern region	19	7.9
North region	65	27.0
Southern region	47	19.5
Qualification (physical therapy)		
Diploma certificate	7	2.9
Bachelor’s degree	164	68.0
Doctor of physical therapy	12	5.0
Master’s degree	29	12.0
Doctoral degree (PhD or equivalent)	29	12.0
Experience		
< 1 year	46	19.1
1–5 years	84	34.9
6–10 years	49	20.3
11–20 years	47	19.5
> 20 years	15	6.2
Working place		
Public hospital/clinic/center	111	46.1
Private hospital/clinic/center	86	35.7
University hospital/clinic/center	39	16.2
Unemployed	5	2.1
Specialization (physical therapy)		
General	133	55.2
Orthopedic	52	21.6
Neurological	12	5.0
Pediatric	18	7.5
Geriatric	5	2.1
Sports	10	4.1
Chest	5	2.1
Other	6	2.5

### Learning sources, barriers, and beliefs toward radiological interpretation

**Fig. 1:**
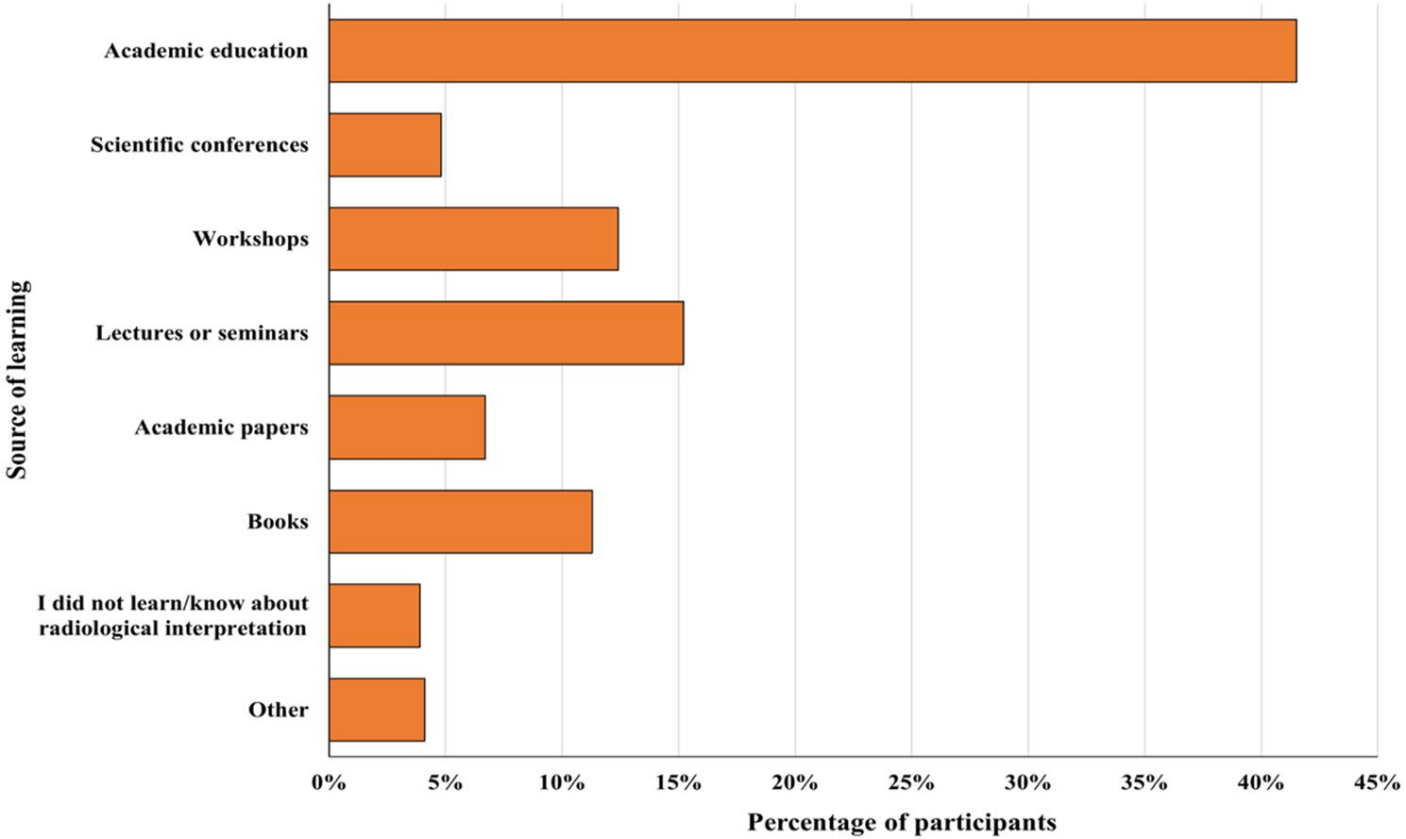
Sources of learning about radiological interpretation reported by participants

As shown in Fig. 1; Table 3, academic education was the most reported source of knowledge (41.5%), followed by lectures or seminars (15.2%), workshops (12.4%), books (11.3%), academic papers (6.7%), and scientific conferences (4.8%). A minority of participants reported not having learned about radiological interpretation (3.9%) or indicated other sources, such as clinical experience, guidance from a radiologist or physician, medical websites, coaching, self-learning, educational videos, or shadowing radiologists (4.1%).

**Table 3:** Survey responses on learning sources, barriers, and beliefs toward radiological interpretation among the participating PTs (*n* = 241)

Question	*n*	%
How did you learn/know about radiological interpretation? *		
Academic education	180	41.5
Scientific conferences	21	4.8
Workshops	54	12.4
Lectures or seminars	66	15.2
Academic papers	29	6.7
Books	49	11.3
I did not learn/know about radiological interpretation	17	3.9
Other	18	4.1
From your perspective, what are the factors that may limit the ability to conduct radiological interpretations effectively? *		
Insufficient education and training	190	40.0
Insufficient access to advanced imaging technology	91	19.2
Poor communication or collaboration among team members	89	18.7
Inadequate time for interpretation (limited staffing/heavy workload)	57	12.0
Technical issues with imaging systems (e.g., poor image quality)	48	10.1
Are you interested in radiological interpretations?		
Yes	216	89.6
No	25	10.4
Would you be interested in attending a lecture or hands-on seminar about radiological interpretation?		
Yes	219	90.9
No	22	9.1

*PTs* Physical Therapists

*Multiple responses were allowed for this item

**Fig. 2:**
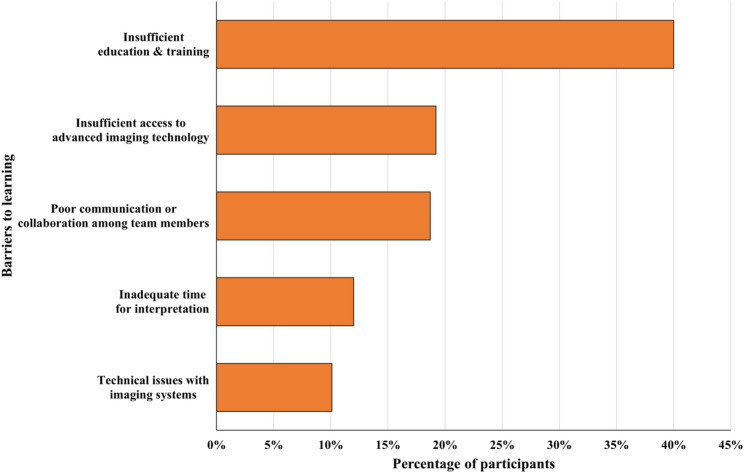
Factors reported by participants as barriers to effective radiological interpretation

The participants identified several barriers that may limit their ability to interpret radiological images effectively, as shown in Fig. 2. The most frequently reported barrier was insufficient education and training (40.0%), followed by a lack of access to advanced imaging technology (19.2%), poor communication or collaboration (18.7%), limited time due to staffing or workload (12.0%), and technical issues related to imaging systems (10.1%). Finally, 89.6% of the participants expressed interest in radiological interpretation, and 90.9% indicated that they would be interested in attending a related lecture or hands-on seminar.

### Contribution frequency and attitudes toward radiograph interpretation

As presented in Table 4, statistically significant differences were observed in participants’ frequency of contribution to radiological interpretation. A total of 46.1% of the participants reported contributing frequently, 30.3% reported contributing sometimes, and 23.7% reported contributing rarely (*p* < 0.005). Pairwise comparisons revealed that those who contributed frequently were significantly more common than those who contributed rarely (*p* < 0.005) or sometimes (*p* = 0.005). No significant difference was found between those who contributed rarely or sometimes (*p* = 0.161).

With respect to attitudes toward the role of PTs in radiograph interpretation, 83.0% agreed that PTs should interpret radiographs, whereas 11.6% were neutral and 5.4% disagreed (*p* < 0.005). Pairwise comparisons indicated that agreement was significantly more common than both disagreement (*p* < 0.005) and neutrality (*p* < 0.005) while neutrality was also significantly more common than disagreement (*p* = 0.019).

**Table 4:** Main differences and pairwise comparisons of contribution frequency and attitudes toward radiograph interpretation (*n* = 241)

Category	*n*	%	*p*-value	Pairwise comparison	*p*-value
Frequency of contribution to radiological interpretation					
Rarely	57	23.7	< 0.005*	Rarely vs. Sometimes	0.161
Sometimes	73	30.3		Rarely vs. Frequently	< 0.005*
Frequently	111	46.1		Sometimes vs. Frequently	0.005*
PTs should interpret radiographs as part of their job					
Disagree	13	5.4	< 0.005*	Disagree vs. Neutral	0.019*
Neutral	28	11.6		Disagree vs. Agree	< 0.005*
Agree	200	83.0		Neutral vs. Agree	< 0.005*

Note: Significant differences were examined via chi-square tests

*Significant difference at α = 0.05

### Factors associated with frequent contributions to radiological interpretation

As shown in Table 5, a significant proportion of both male (48.6%, *p* < 0.005) and female (43.9%, *p* = 0.032) participants reported frequently contributing to radiological interpretation. Regionally, participants from the Western (53.8%, *p* = 0.018) and Northern (47.7%, *p* = 0.041) regions demonstrated significantly high proportions of frequent contributions, whereas those from the Central, Eastern, and Southern regions did not show statistically significant percentages. Regarding qualifications, a significant proportion of bachelor’s degree holders (50.0%, *p* < 0.005) reported frequent contributions. In contrast, participants with diplomas, doctor of physical therapy, master’s degrees, or doctoral degrees did not report statistically significant proportions of frequent contributions.

Years of experience were also associated with contribution frequency. Participants with 1–5 years (46.4%, *p* = 0.039) and 6–10 years (51.0%, *p* = 0.004) of experience showed significant levels of frequent contribution, whereas those with less than 1 year, 11–20 years, or more than 20 years of experience did not. In terms of workplace, significant proportions of participants working in public (52.3%, *p* < 0.005) and private (46.5%, *p* = 0.034) settings reported frequent contributions. However, those employed at university-affiliated facilities or unemployed did not report significant proportions. Among areas of specialization, only participants practicing in general physical therapy reported a significant level of frequent contribution (47.4%, *p* = 0.002). The proportions of other specialties were not significantly different.

**Table 5:** Associations of demographic and professional factors with frequent contributions to radiological interpretation (*n* = 111)

Characteristics		Proportion of participants who frequently contributed to radiological interpretation		
		*n*	%	*p*-value
Sex	Male	53	48.6	< 0.005*
	Female	58	43.9	0.032*
Practicing region in Saudi Arabia	Central	28	39.4	0.546
	Western	21	53.8	0.018*
	Eastern	9	47.4	0.368
	Northern	31	47.7	0.041*
	Southern	22	46.8	0.067
Qualification	Diploma certificate	1	14.3	0.102
	Bachelor’s degree	82	50.0	< 0.005*
	Doctor of physical therapy	5	41.7	0.779
	Master’s degree	12	41.4	0.639
	Doctoral degree (PhD or equivalent)	11	37.9	0.786
Experience	< 1 year	19	41.3	0.345
	1–5 years	39	46.4	0.039*
	6–10 years	25	51.0	0.004*
	11–20 years	21	44.7	0.240
	> 20 years	7	46.7	0.247
Working place	Public hospital/clinic/center	58	52.3	< 0.005*
	Private hospital/clinic/center	40	46.5	0.034*
	University hospital/clinic/center	12	30.8	0.794
	Unemployed	1	20.0	0.449
Primary area of specialization in physical therapy	General	63	47.4	0.002*
	Orthopedic	24	46.2	0.138
	Neurological	5	41.7	0.779
	Pediatric	7	38.9	0.846
	Geriatric	1	20.0	0.819
	Sports	6	60.0	0.527
	Chest	3	60.0	0.449
	Other	2	33.3	0.607

Note: Significant differences were examined via chi-square tests

*Significant difference at α = 0.05

### Factors associated with agreement with PTs interpreting radiographs

As shown in Table 6, a significant proportion of both male (84.4%, *p* < 0.005) and female (81.8%, *p* < 0.005) participants agreed that PTs should interpret radiographs as part of their professional role. Regionally, significant proportions of agreement were observed among participants from all five regions: western (89.7%, *p* < 0.005), southern (87.2%, *p* < 0.005), northern (83.1%, *p* < 0.005), central (78.9%, *p* < 0.005), and eastern (73.7%, *p* = 0.001) regions. With respect to qualifications, a significant proportion of bachelor’s degree holders (82.9%, *p* < 0.005) and participants with master’s (89.7%, *p* < 0.005) or doctoral degrees (82.8%, *p* < 0.005) agreed that PTs should interpret radiographs. In contrast, agreement was not statistically significant among participants with a diploma or doctor of physical therapy degree.

Work experience was also associated with agreement levels. A significant proportion of participants with less than 1 year (78.3%, *p* < 0.005), 1–5 years (79.8%, *p* < 0.005), 6–10 years (87.8%, *p* < 0.005), 11–20 years (85.1%, *p* < 0.005), or more than 20 years of experience (93.3%, *p* = 0.001) agreed that PTs should interpret radiographs. With respect to workplace setting, significant agreement was observed among participants working in public (87.4%, *p* < 0.005), private (80.2%, *p* < 0.005), and university hospitals (76.9%, *p* < 0.005). In terms of specialization, significant proportions of agreement were found among those in general (82.0%, *p* < 0.005), orthopedic (86.5%, *p* < 0.005), pediatric (83.3%, *p* < 0.005), neurological (75.0%, *p* = 0.009), and sports (90.0%, *p* = 0.011) specialties. All participants in the chest and “other” categories (100%) agreed with PTs interpreting radiographs.

**Table 6:** Associations of demographic and professional factors with agreement with PTs interpreting radiographs (*n* = 200)

Characteristics		Proportion of participants who agree that PTs should interpret radiographs		
		n	%	*p*-value
Sex	Male	92	84.4	< 0.005*
	Female	108	81.8	< 0.005*
Practicing region in Saudi Arabia	Central	56	78.9	< 0.005*
	Western	35	89.7	< 0.005*
	Eastern	14	73.7	0.001*
	North	54	83.1	< 0.005*
	Southern	41	87.2	< 0.005*
Qualification	Diploma certificate	5	71.4	0.257
	Bachelor’s degree	136	82.9	< 0.005*
	Doctor of physical therapy	9	75.0	0.083
	Master’s degree	26	89.7	< 0.005*
	Doctoral degree (PhD or equivalent)	24	82.8	< 0.005*
Experience	< 1 year	36	78.3	< 0.005*
	1–5 years	67	79.8	< 0.005*
	6–10 years	43	87.8	< 0.005*
	11–20 years	40	85.1	< 0.005*
	> 20 years	14	93.3	0.001*
Working place	Public hospital/clinic/center	97	87.4	< 0.005*
	Private hospital/clinic/center	69	80.2	< 0.005*
	University hospital/clinic/center	30	76.9	< 0.005*
	Unemployed	4	80.0	0.180
Primary area of specialization in physical therapy	General	109	82.0	< 0.005*
	Orthopedic	45	86.5	< 0.005*
	Neurological	9	75.0	0.009*
	Pediatric	15	83.3	< 0.005*
	Geriatric	2	40.0	0.655
	Sports	9	90.0	0.011*
	Chest	5	100.0	Constant
	Other	6	100.0	Constant

Note: Significant differences were examined via chi-square tests

*Significant difference at α = 0.05

### Relationships among practice, beliefs, and engagement with radiological interpretation

Table 7 presents the results of the binary logistic regression examining the relationships among contribution frequency, attitudes, and participants’ interest in radiological interpretation as well as their willingness to attend educational activities. When participants who frequently contributed to radiological interpretation were used as the reference group, those who rarely contributed were significantly more likely to express interest in radiological interpretation (OR = 4.0, *p* = 0.007). However, no significant difference in interest was observed among participants who contributed occasionally (*p* = 0.621). Similarly, participants who contributed rarely or occasionally did not show a significant association with interest in attending a lecture or seminar about radiological interpretation (*p* = 0.192 and 0.265, respectively).

With respect to attitudes and the use of participants who disagreed with the role of PTs in interpreting radiographs as a reference, those who agreed were approximately seven times more likely to express interest in radiological interpretation (OR = 7.6, *p* = 0.003). Furthermore, the likelihood of expressing interest in attending a lecture or hands-on seminar was also significant among those who agreed (OR = 5.7, *p* = 0.018). No significant associations were observed for the neutral group regarding interest in radiological interpretation or attendance at educational activities (*p* = 0.756 and 0.554, respectively).

The results of the sensitivity analysis using the alternative coding schemes confirmed the magnitude and direction of key relationships (Appendix B.). For frequency of contribution, the effect of rare contribution on interest in radiological interpretation remained elevated and in the same direction (OR = 2.4), although with a slightly higher *p*-value (*p* = 0.062). For attitudes toward radiograph interpretation, participants who agreed that PTs should interpret radiographs also expressed a high interest in radiological interpretation (OR = 8.9, *p* < 0.005). A similar pattern was observed for interest in attending a lecture, where the association remained high and robust (OR = 7.9, *p* < 0.005).

**Table 7:** Logistic regression analysis for the relationships among contribution frequency, beliefs, and interest in radiological interpretation and education (*n* = 241)

Attribute	Interested in radiological interpretations				Interested in attending a lecture or seminar on radiological interpretation			
	Yes (%)	No (%)	OR (95% CI)	*p*-value	Yes (%)	No (%)	OR (95% CI)	*p*-value
Frequency of contribution to radiological interpretation								
Rarely	45 (78.9%)	12 (21.1%)	4.0 (1.5–10.7) *	0.007	50 (87.7%)	7 (12.3%)	0.5 (0.2–1.4)	0.192
Sometimes	67 (91.8%)	6 (8.2%)	1.3 (0.4–4.1)	0.621	65 (89.0%)	8 (11.0%)	0.5 (0.2–1.6)	0.265
Frequently	104 (93.7%)	7 (6.3%)	1	-	104 (93.7%)	7 (6.3%)	1	-
PTs should interpret radiographs as part of their job								
Disagree	9 (69.2%)	4 (30.8%)	1	-	10 (76.9%)	3 (23.1%)	1	-
Neutral	18 (64.3%)	10 (35.7%)	0.8 (0.2–3.3)	0.756	19 (67.9%)	9 (32.1%)	0.6 (0.1–2.9)	0.554
Agree	189 (94.5%)	11 (5.5%)	7.6 (2.0-28.7) *	0.003	190 (95.0%)	10 (5.0%)	5.7 (1.4–24.0) *	0.018

*OR* odds ratio, *CI* confidence interval

*Significant at α = 0.05

## Discussion

This study provides a comprehensive national perspective from Saudi Arabia on PTs’ engagement, attitudes, and educational interests in radiological interpretation. It addresses a notable gap in the literature regarding the extent to which PTs actively engage in interpreting radiological images and their perceptions of their role in this area. This shows that the majority of PTs (83%) feel that interpreting radiographs should be part of their professional responsibilities. Nearly half of the respondents (46%) reported some level of involvement in radiological interpretation, though the specific activities and their clinical impact warrant further clarification. Additionally, more than 90% indicated that they would be interested in receiving additional training on the subject. The findings demonstrate a significant level of engagement and notable interest in radiological interpretation.

Despite academic education being the most frequently reported source of knowledge (41.5%), the most cited barrier to effective radiological interpretation was insufficient education and training (40.0%). This reveals a gap between academic education and the practical training required for radiological reasoning in clinical practice. While academic programs may cover theoretical aspect of radiological assessment, they often provide limited opportunities for students to apply these concepts to real patients’ cases [23, 24]. Previous studies have similarly reported that many PTs feel uncertain about when diagnostic imaging is appropriately indicated and how to interpret imaging findings within their clinical scope. These difficulties were primarily attributed to inadequate formal training (32%). Additionally, 69% of PTs reported that they require mentorship and support from other professionals [7].

In Saudi Arabia, PTs’ ability to make independent radiological decisions may be restricted by institutional or regulatory constraints [4, 8]. This discrepancy between formal education and the competencies required in practice, highlights the need to revise curricula to align with current clinical standards [14]. For instance, a recent Delphi study indicated that PTs in critical care are expected to understand pathophysiology, interpret radiographs, and carry out functional assessments [25], Currently, physical therapy curricula provide only limited and variable training in diagnostic and procedural imaging. While some programs include imaging education, the scope, depth, and integration of such training differ considerably between institutions, and there is no consistent standard across curricula [14, 23]. This variability highlights the need for more consistent and comprehensive imaging education to better prepare PTs for evolving clinical roles. This could include the introduction of modules on radiological evaluation, interdisciplinary teaching approaches, along with practical workshops on imaging interpretation during clinical rotations.

The high level of interest (89.6%) and willingness (90.9%) to attend educational activities related to radiological interpretation suggest strong motivation to enhance competencies in this area. However, an interesting and inconsistent finding revealed that PTs who rarely contributed to radiological interpretation were significantly more likely to express interest in further training than those who frequently contributed (OR = 4.0, *p* = 0.007). This may indicate a lack of confidence, limited institutional support or access to imaging systems, or legal and professional barriers rather than a lack of motivation [8]. These findings point to the utility of personalized and flexible educational approaches such as modular training, case-based learning or mentorship-driven models, especially for under-engaged practitioners [26]. Furthermore, the use of digital platforms and simulation technologies such as 3D virtual reality [27], or real-time interactive X-ray simulation [28] in clinical education may facilitate learning, promote self-paced engagement and improve diagnostic reasoning skills [29]. These strategies can also help address geographic differences by offering remote learning opportunities, especially for practitioners in rural regions.

The frequency of contribution to radiograph interpretation was significantly associated with several factors, including sex, region, educational level, years of experience, workplace setting, and area of specialization. Mid-career professionals (with 1–10 years of experience) and those working in public or private settings were more likely to contribute frequently, possibly reflecting greater clinical responsibility and routine exposure to imaging. Notably, general physical therapy was the only specialty significantly associated with frequent contributions, suggesting a potential need for greater imaging exposure and training in other subspecialties, such as neurology and pediatrics, where imaging interpretation may still be limited or delegated. This is consistent with previous studies showing that PTs with an orthopedic caseload of 50% or more (*p* < 0.001) and those working in the private sector (*p* < 0.001) demonstrated greater interest in ordering radiographs [7].

The attitudes toward radiograph interpretation were positive, with 83.0% of the participants agreeing that PTs are interested in radiograph interpretation. This agreement was consistently distributed across different regions, qualifications, and levels of experience, reflecting a growing interest in the evolving diagnostic contribution of PTs within interprofessional care teams. Notably, PTs who supported this role were significantly more likely to express interest in training and active engagement, highlighting an empirically supported link between professional attitudes and motivation for skill development.

To translate this interest into safe and effective clinical practice, national stakeholders, including professional bodies and educational institutions, should prioritize the integration of imaging competencies into undergraduate and postgraduate curricula such as blended learning and simulation. This integration could follow competency-based education that define measurable learning outcomes [30], such as understanding imaging modalities, applying clinical decision rules, and interpreting common radiological findings. Blended learning approaches, combining online theoretical modules with face-to-face practical workshops, can enhance accessibility and accommodate diverse learning needs. Furthermore, simulation-based education can provide realistic, risk-free environments for experimental learning and skill improvement [31].

Additionally, there is a clear need for continuing professional development programs that are accessible and standardized, ensuring upskilling across the profession. Developing clear guidelines on scope of practice and legal responsibilities can further support PTs in safely applying imaging interpretation within clinical settings. Future research should focus on the design, implementation and evaluation of educational interventions that address the training gap, particularly for underrepresented subgroups. It is also important to investigate how PTs’ interpretative skills influence referral patterns, cost effectiveness, and patient safety to support broader and systemic changes in training and practice policies.

Despite its valuable findings, this study has several limitations that should be considered. First, the use of convenience sampling may limit the generalizability of our findings to the broader physical therapy workforce in Saudi Arabia. Second, as a cross-sectional study, it can identify associations but cannot establish causal relationships between the examined factors and PTs’ attitudes or involvement in radiological interpretation. Although associations were found, it is impossible to determine whether these relationships are directional or temporal. However, the study offers important baseline data that identify practice patterns and potential research areas. To address these limitations, future research should aim to include larger samples and implement the longitudinal or experimental research designs to explore causal pathways. Additionally, validation methods such as comparing self-reported practices with direct observation, chart audits, or case-based performance assessments could strengthen future evaluations of PTs’ imaging related competencies. Finally , the measure of *“engagement with radiological information”* did not distinguish between different forms of involvement—such as collaborative discussion of imaging findings, integration of radiology reports into clinical reasoning, or informal reference to imaging during patient management. Consequently, the reported frequencies may reflect a range of engagement rather than distinct levels of interpretive responsibility. Future studies should examine these dimensions more precisely, using objective methods such as structured case vignettes, scenario-based assessments, or observational designs to clarify the nature and extent of PTs’ engagement with radiological information.

## Conclusion

This study highlights the growing interest and positive attitudes of PTs in Saudi Arabia toward radiological interpretation, despite the notable gap between academic preparation and clinical demands. While many PTs report academic education as their primary source of radiological knowledge, inadequate training remains a key barrier to effective clinical application. The findings emphasize the need to update physical therapy curricula, expand access to continuing professional development, and establish clear professional guidelines to support the safe and effective integration of imaging into practice. Addressing these areas may improve clinical decision-making and diagnostic accuracy and empower PTs to meet the evolving demands of modern healthcare systems.

## Supplementary Information

Supplementary Material 1: Appendix A. PDF (Study questionnaire).

Supplementary Material 2: Appendix B. PDF (Results of sensitivity analysis).

## Data Availability

The data supporting the findings of this study are available from the corresponding author upon reasonable request.

## Abbreviations

SPSS: Statistical Package for the Social Sciences
PT: Physical Therapist
