# Supplementary material for: Perspectives of Physical Therapists in Saudi Arabia on radiological interpretation: attitudes, engagement, and educational needs
Source: BMC Med Educ. 2025 Dec 2;26:33. doi: 10.1186/s12909-025-08367-1 (PMC12781575; doi:10.1186/s12909-025-08367-1)
Supplement: Supplementary file 1 — Supplementary Material 1: Appendix A. PDF (Study questionnaire). [file 12909_2025_8367_MOESM1_ESM.pdf]

Physical therapists' ability to interpret radiographs

Please read the following information carefully.

Purpose of the study: This study aims to evaluate the skills of Physical therapists in interpreting different radiological cases.

You are eligible to participate in this survey if you meet the following criteria: physical therapy certificate holder

Do I have to take part? Your participation in this study is voluntary and you can withdraw at any time.

Risks of being a participant: There are no foreseeable risks of being a participant in an online survey. This survey will not ask you about any identity. The data collected will be used for research purposes only, and may be presented at academic conferences or published in scientific journals

Benefits of being a participant: You may not receive direct benefit from participating in this study, but we anticipate the study findings will help us to better understand the current knowledge of physical therapists in evaluating radiographs.

Time expected to complete this survey: 3 to 5 minutes

Ethical approval number: UT-505-313-2025

Privacy & confidentiality: Your privacy will be maintained at all times, and you will not be identified and your data will be securely stored. If you have any questions about this research, please contact Researchers at salamrani@ut.edu.sa.

Do you agree to participate in this study? *

- ☐ Yes
- ☐ No

General information

Please select your gender*

- ☐ Male
- ☐ Female

Please select your age group *

- ☐ 22-29
- ☐ 30-39
- ☐ 40-49
- ☐ 50-59
- ☐ 60 and above

Please select the region that you work in.*

- ☐ Northern region
- ☐ Southern region
- ☐ Central region
- ☐ Eastern region
- ☐ Western region

Please select the type of health care facility you work in.*

- ☐ Public hospital/ centers/ clinics
- ☐ University hospital/centers/ clinics
- ☐ Private hospital /centers/ clinics
- ☐ Other:

Please select your years of clinical experience. *

- ☐ Less than one year
- ☐ 1 – 5 years
- ☐ 6 – 10 years
- ☐ 11 – 20 years
- ☐ More than 20 years

Please select your highest earned academic degree. *

- ☐ Diploma
- ☐ Bachelor Degree
- ☐ Doctor of Physical Therapy (DPT)
- ☐ Master Degree
- ☐ Doctoral (PhD) Degree

Please select your area of specialization in physical therapy.*

- ☐ General
- ☐ Orthopedic
- ☐ Neurological
- ☐ Pediatric
- ☐ Geriatric
- ☐ Sports
- ☐ Other:

Radiological assessment

How often do you contribute in radiological interpretation?*

- ☐ Always
- ☐ Often
- ☐ Sometimes
- ☐ Rarely
- ☐ Never

How did you learn /know about radiological interpretation? (*Multiple responses are possible.)*

- ☐ Academic education
- ☐ Scientific conference
- ☐ Workshop
- ☐ Lecture or seminar
- ☐ Academic paper
- ☐ Book
- ☐ I did not learn/know about radiological interpretation
- ☐ Other:

From your perspective, what are the factors that may limit the ability to conduct radiological interpretations effectively?*

- ☐ Insufficient education and training
- ☐ Insufficient access to advanced imaging technology
- ☐ Poor communication or collaboration among team members
- ☐ Inadequate time for interpretation (limited staffing/ heavy workload)
- ☐ Technical issues with imaging systems (e.g. Poor image quality)

Do you think physiotherapists should interpret radiographs as part of their practice/job?*

- ☐ Strongly agree
- ☐ Agree
- ☐ Neutral
- ☐ Disagree
- ☐ Strongly disagree

Are you interested in radiological interpretations?*

- ☐ Yes
- ☐ No

Would you be interested in attending a lecture or hands-on seminar about radiological interpretation?*

- ☐ Yes
- ☐ No
