# Supplementary material for: Perspectives of Physical Therapists in Saudi Arabia on radiological interpretation: attitudes, engagement, and educational needs
Source: BMC Med Educ. 2025 Dec 2;26:33. doi: 10.1186/s12909-025-08367-1 (PMC12781575; doi:10.1186/s12909-025-08367-1)
Supplement: Supplementary file 2 — Supplementary Material 2: Appendix B. PDF (Results of sensitivity analysis). [file 12909_2025_8367_MOESM2_ESM.pdf]

Appendix B. Sensitivity analysis of logistic regression models for the relationship between contribution frequency, beliefs, and interest in radiological interpretation and education (n = 241)

Attribute	Interested in radiological interpretations				Interested in attending a lecture or seminar on radiological interpretation			
	Yes (%)	No (%)	OR (95% CI)	*p*-value	Yes (%)	No (%)	OR (95% CI)	*p*-value
**Frequency of contribution to radiological interpretation**								
**Rarely**	112 (86.2%)	18 (13.8%)	2.4 (1.0-5.9)	0.062	115 (88.5%)	15 (11.5%)	1.9 (0.8-4.9)	0.166
**Frequently**	104 (93.7%)	7 (6.3%)	1	-	104 (93.7%)	7 (6.3%)	1	-
**Physical therapists should interpret radiographs as part of their job**								
**Disagree**	27 (65.9%)	14 (34.1%)	1	-	29 (70.7%)	12 (29.3%)	1	-
**Agree**	189 (94.5%)	11 (5.5%)	8.9 (3.7-21.6)*	< 0.005	190 (95.0%)	10 (5.0%)	7.9 (3.1-19.8)*	< 0.005

OR, odds ratio; CI, confidence interval
*Significant at $\alpha = 0.05$
